# Draft Genome Sequence of the Crude Oil-Degrading and Biosurfactant-Producing Strain *Cobetia* sp. QF-1

**DOI:** 10.1128/genomeA.01456-17

**Published:** 2018-01-18

**Authors:** Ping Guo, Binxia Cao, Xue Qiu, Jianguo Lin

**Affiliations:** a College of Environmental Science and Engineering, Dalian Maritime University, Dalian, China

## Abstract

We report here the draft genome of *Cobetia* sp. QF-1, a cold-adapted bacterium isolated from crude oil-contaminated seawater of the Yellow Sea, China. This genome is approximately 4.1 Mb (G+C content, 57.44%) with 3,513 protein-coding sequences. *Cobetia* sp. QF-1 shows crude oil degradation and biosurfactant production activity at low temperature.

## GENOME ANNOUNCEMENT

The *Cobetia* genus is 1 of 11 genera of the family *Halomonadaceae* and is most closely related to members of the genus *Halomonas* (1). The type strain has been described as aerobic, Gram-negative, motile, rod-shaped, and slightly halophilic (2). *Cobetia* sp. QF-1 can degrade crude oil and produce biosurfactants at 0°C. Although some isolated marine bacteria can degrade oil efficiently around 0°C, most of them have been isolated from polar environments (3, 4); a few have been isolated from China. Several genomes of *Cobetia* strains have been sequenced before (5–7), but a marine *Cobetia* strain from China with the capacity to degrade hydrocarbons and produce biosurfactants at 0°C is lacking. Here, we report the draft genome sequence of *Cobetia* sp. QF-1, isolated from the seawater of Yellow Sea, China.

The genome sequencing of *Cobetia* sp. QF-1 was performed using the Illumina HiSeq platform at NovogenBio, Beijing, China. Reads were assembled using SOAPdenovo software version 2.21 (8). Protein-coding sequences were functionally annotated with the Clusters of Orthologous Groups of Proteins (COG) (9), Gene Ontology (GO) (10), and KEGG databases (11). tRNAs and rRNAs were detected using tRNAscan-SE (12) and rRNAmmer software (13), respectively.

The genome of *Cobetia* sp. QF-1 was 4,084,184 bp with a 57.44% GC content. The genome contained 3,513 predicted protein-coding sequences, and the total length of the genes was 3,470,121 bp, which accounted for 84.96% of the genome. The genome also encoded 71 tRNAs and 5 rRNAs.

According to the annotation results, many genes involved in petroleum hydrocarbon degradation, biosurfactant production, and cold adaption were found in the genome of *Cobetia* sp. QF-1. This draft genome sequence of *Cobetia* sp. QF-1 will help in understanding the genetic basis of crude oil degradation and biosurfactant production by *Cobetia* spp.

### Accession number(s).

This whole-genome shotgun project has been deposited at DDBJ/ENA/GenBank under the accession number NBVS00000000. The version described in this paper is the first version, NBVS01000000.
